# A Comprehensive, Three-Dimensional Analysis of a Large-Scale, Multi-Fuel, CFB Boiler Burning Coal and Syngas. Part 2. Numerical Simulations of Coal and Syngas Co-Combustion

**DOI:** 10.3390/e22080856

**Published:** 2020-07-31

**Authors:** Jaroslaw Krzywanski, Karol Sztekler, Mateusz Szubel, Tomasz Siwek, Wojciech Nowak, Łukasz Mika

**Affiliations:** 1 Faculty of Science and Technology, Jan Dlugosz University in Czestochowa, al.Armii Krajowej 13/15, 42-200 Czestochowa, Poland; 2 Faculty of Energy and Fuels, AGH University of Science and Technology, al. Mickiewicza 30, 30-059 Krakow, Poland; sztekler@agh.edu.pl (K.S.); mzubel@agh.edu.pl (M.S.); siwek@agh.edu.pl (T.S.); wnowak@agh.edu.pl (W.N.); lmika@agh.edu.pl (Ł.M.)

**Keywords:** circulating fluidized bed, CFD, multi-fuels boilers, modeling, simulation, co-combustion

## Abstract

This paper presents the results of numerical computations for a large-scale OFz-425 CFB (circulating fluidized bed) boiler utilizing coal and syngas. Four different operating scenarios are considered, including the reference variant, corresponding to the conventional, mono-combustion of bituminous coal, and three tests involving replacement of secondary air and part of the coal stream with syngas fed by start-up burners. Pressure, gas velocity, temperature, and carbon dioxide distribution in the combustion chamber are discussed in the paper. The results indicate that the syngas supply leads to an increase in local temperature and carbon dioxide concentrations. The proposed concept is not advisable as it may lead to frequent emergency stops of the CFB boiler.

## 1. Introduction

The performance of engineering devices is degraded by the presence of irreversibilities, and entropy generation constitutes their measure [1]. Since entropy generation is used for establishing criteria for the performance of engineering devices, the irreversibilities associated with a process should be reduced in order to increase engine performance [1,2]. Analysis and optimization via modeling can lead to an increase in a system’s performance [2,3,4]. Computational fluid dynamics (CFD) models are considered to be the most comprehensive due to 3D, detailed consideration of chemical kinetics and individual physical processes, gas and solids continuity formulas, as well as momentum balances, and appropriate constitutive equations [5]. The CFD models can be used for complex analysis of multi-fuel CFB (circulating fluidized bed) unit operation concepts [6,7,8,9]. Various 3D-CFB models can be found in the literature [10,11,12,13,14,15], some of which consider the multi-fuel, CFB concept; however, the use of gaseous fuel, with a properly organized gas supply system, cannot be found in said literature. Such an idea may be beneficial for a power unit’s flexibility and provides the stable operation of a boiler furnace with a substantial demand reduction in its capacity.

On the other hand, acceptable boiler design parameters may be exceeded due to changes in flue gas velocity and temperature profiles. Such cases may result in the destruction of some boiler components or a reduction in their service life [16,17,18,19].

The generated CFD model described in Part I of this paper was applied to study the influence of multi-fuel coal and syngas combustion on some operation issues of the large scale OFz-425 CFB boiler.

To the best of our knowledge, there are no reports in pertinent literature regarding comprehensive 3D-CFD modeling of a large-scale, multi-fuel, CFB boiler, burning coal and syngas.

## 2. Materials and Methods

The model was developed in the ANSYS Workbench environment for Fluent Solver. The two-phase, 3D model of homogenous and heterogeneous combustion employs CFD methods for the flow of a reactive mixture. The equations for mass, enthalpy, momentum, and selected gaseous components relevant to the combustion process were applied using the finite volume method. Reynolds-averaged Navier–Stokes equations (RANS) with the k-omega BSL turbulence model were used in the study. The heat transfer between the bed and the combustion chamber walls was considered using convection and radiation models.

Computations and validation of the model were successfully performed on data from an existing, large-scale, two-pass OFz-425 CFB boiler at 100% load, produced by RAFAKO S.A., Poland. The main parts of the boiler are the riser with the combustion chamber made of membrane-walls, superheater II (SH II), reheater II (RH II), cyclones, and the second pass in the convection cage, consisting of built-in superheaters and reheaters, as well as an economizer and a rotary air heater.

The dimensions of the cross-sectional area of the combustion chamber in its lower part, immediately above the grid, are 4.6 m × 11.6 m and 8.6 m × 11.6 m, at the height of 6.5 m above the gas distributor level. The total height of the combustion chamber is 37.2 m. Two outlet windows, each with a dimension of 5.7 m × 2.0 m, are located in the upper part of the furnace at a level of 38 m.

Four different operating scenarios were considered in this study. The first (Variant K0) was the reference case, which corresponded to the conventional mono-combustion of bituminous coal. The other three tests corresponded to cases with the replacement of secondary air and part of the coal stream with syngas, fed by start-up burners, as follows:

1. Variant K1 corresponds to the use of Nozzle No. 1, with a total 4.6 kg/s of syngas supplied through two side, start-up burners,
2. Variant K2 considers the simultaneous use of Nozzles No. 1 and No. 2, with a total of 9.2 kg/s of syngas supplied to the combustion chamber through four side, start-up burners, and
3. Variant K3 matches the simultaneous use of Nozzles No’s. 1, 2 and 3, with a total of 13.8 kg/s of syngas supplied to the combustion chamber through four side and two front, start-up burners.

A detailed description of the methodology used in this study can be found in Part I.

## 3. Results

### 3.1. Validation

**Figure 1:**
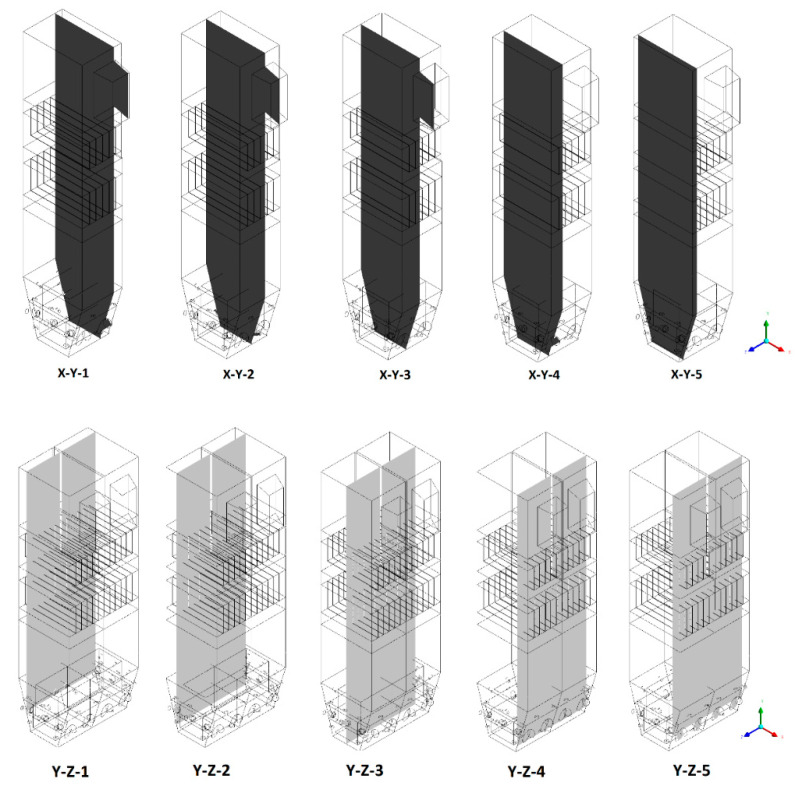
Visualization of sectional views used in the study.

Various methods available within the ANSYS CFD-Post post-processor module are used to conduct the calculations. Selected sections were generated in two perpendicular planes: X-Y and Y-Z (Figure 1). The results are presented in cuts, as follows:

1. Sectional view X-Y parallel to the model’s plane of symmetry, cut X-Y-1, 1.2 m away from the plane of symmetry, then subsequent cuts every 1 m,
2. Sectional view Y-Z parallel to the front/rear wall of the boiler, cut Y-Z-1 1.26 m away from the front wall, then cuts arranged accordingly: 1.76 m; 4.26 m (at equal distance from the front and rear wall), 6.76 m and 7.26 m from the front wall.

Pressure, velocity, temperature, and CO_2_ concentration profiles in the combustion chamber are discussed in the paper. The results are depicted successively for all considered variants of a given cut.

Calculations by model and accessible experimental pressures and temperatures are compared in Table 1 and Table 2. Based on measured data, it can be stated that high predictive accuracy was achieved by the model. The maximum relative error is below 7%.

Such performed CFD models are used for further calculations. As the measured data are mostly confidential, the paper shows a limited number of measuring points located in the lower part of the combustion chamber (Table 1 and Table 2).

However, because they were all located in the most computationally demanding, dense fluidized bed zone, which is additionally the main focus in the present study, the reported error shows the high accuracy of the CFD model [20].

### 3.2. Pressure Distribution in the Combustion Chamber

**Figure 2:**
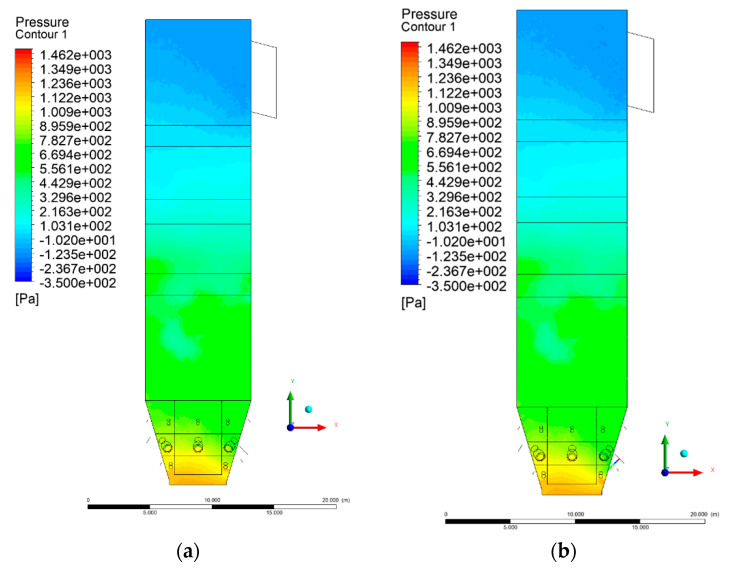
Pressure distribution, sectional view X-Y-3, variants K0 (**a**) and K1 (**b**).

**Figure 3:**
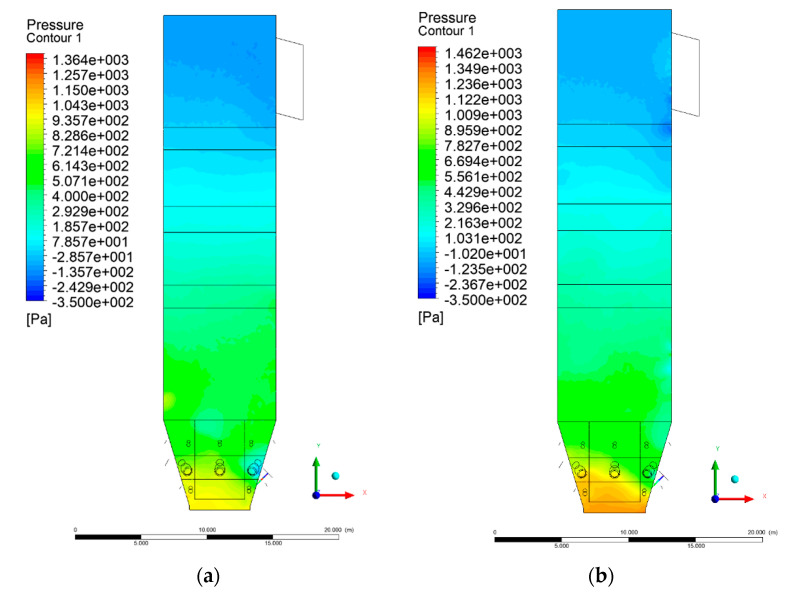
Pressure distribution, sectional view X-Y-3, variants K2 (**a**), K3 (**b**).

Figure 2 and Figure 3 show pressure distribution in the X-Y-3 section. Some discrepancies can be found in the lower part of the wind box. Experimental data indicated a pressure ~2.7 kPa at a height of 1.2 m, while a calculated value, averaged over the cross-sectional domain area, was ~1.4 kPa. This discrepancy results from the simplifications made for the primary air inlet, which were beneficial for shortening the computational time.

It should be noted that the boundary condition for the primary air inlet as the mass flow source was assumed, corresponding to a uniform mass flow over the whole cross-sectional area. The assumed uniform particle size distribution of inert material and fuel also influenced the obtained results. In the upper part of the riser (level 6 m and above), clear analogies to the experimental data can be found. At level 6 m, the pressure averaged over the cross-sectional area was equal to 682 Pa.

**Figure 4:**
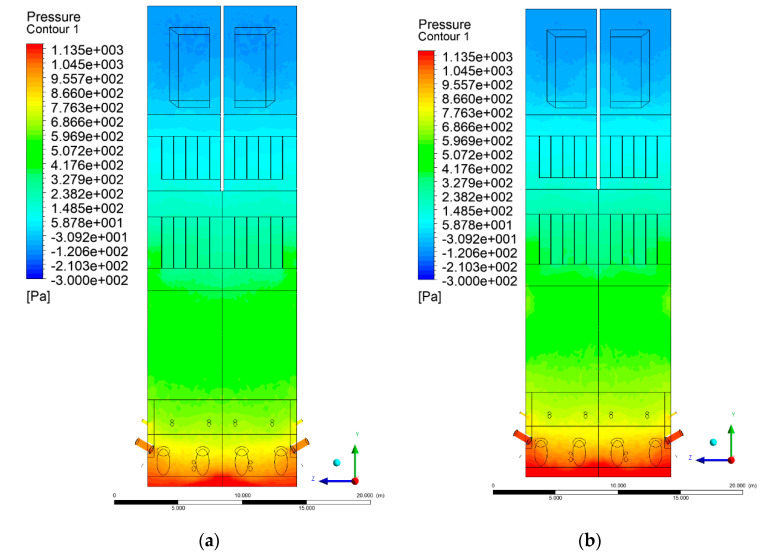
Pressure distribution, sectional view Y-Z-3, variants K0 (**a**), K1 (**b**).

**Figure 5:**
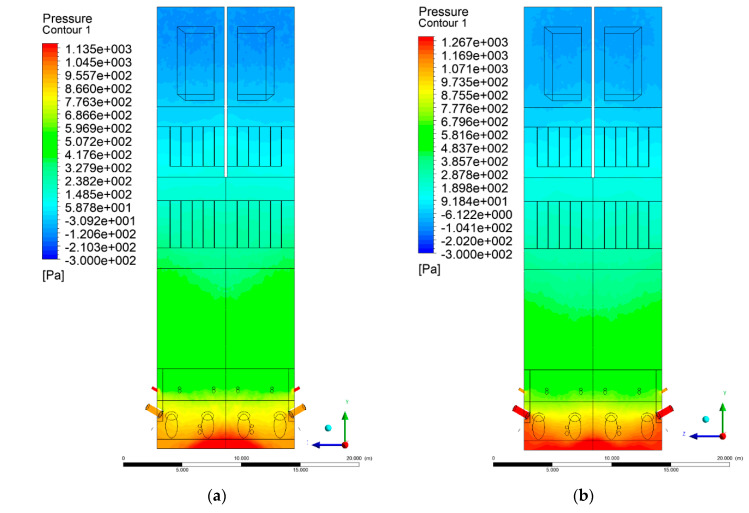
Pressure distribution, sectional view Y-Z-3, variants K2 (**a**), K3 (**b**).

The situation is also similar in the case of the exit zone of the combustion chamber, where, analogously to the experiment, negative pressures from −130 to −350 Pa are registered. Again, it should be emphasized that due to the simplified approach, there is a homogeneous pressure field on the outlet surface of the computational domain. As expected, pressure distribution differed slightly, depending on the considered variant, which was visible on Y-Z sectional views (Figure 4 and Figure 5). These differences are related to the defined syngas properties supplied to the furnace, as well as the applied radiation model, assuming the absorption of radiation by gas components (carbon dioxide, water vapor, methane).

### 3.3. Velocity Distribution in the Combustion Chamber

**Figure 6:**
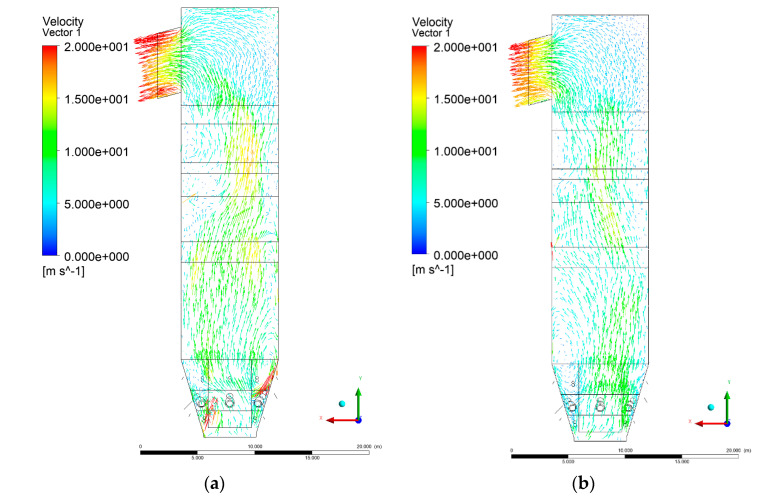
Gas velocity profiles, sectional view X-Y-3, (**a**) Variant K0, (**b**) Variant K1.

**Figure 7:**
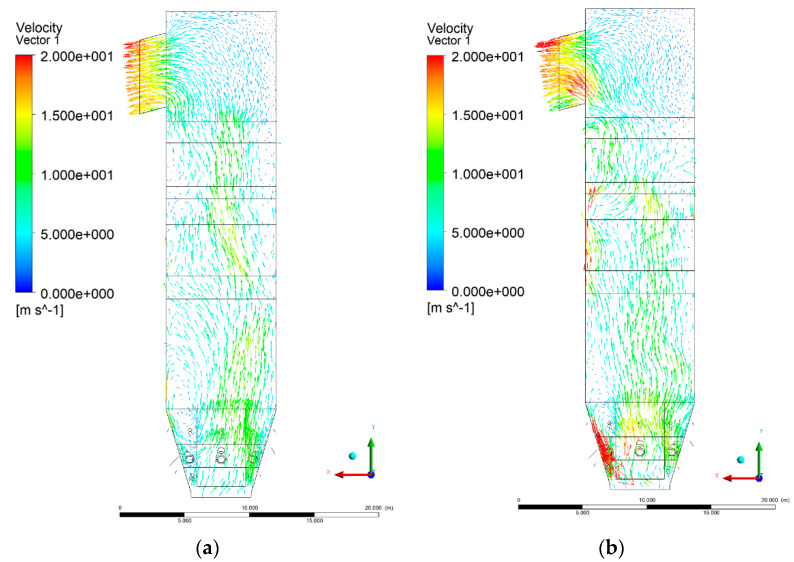
Gas velocity profiles, sectional view X-Y-3, (**a**) Variant K2, (**b**) Variant K3.

**Figure 8:**
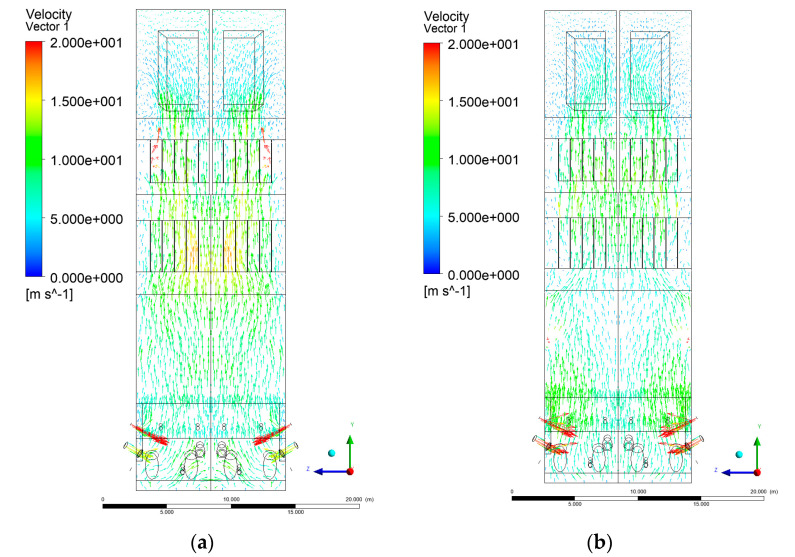
Gas velocity profiles, sectional view Y-Z-3, (**a**) Variant K0, (**b**) Variant K1.

**Figure 9:**
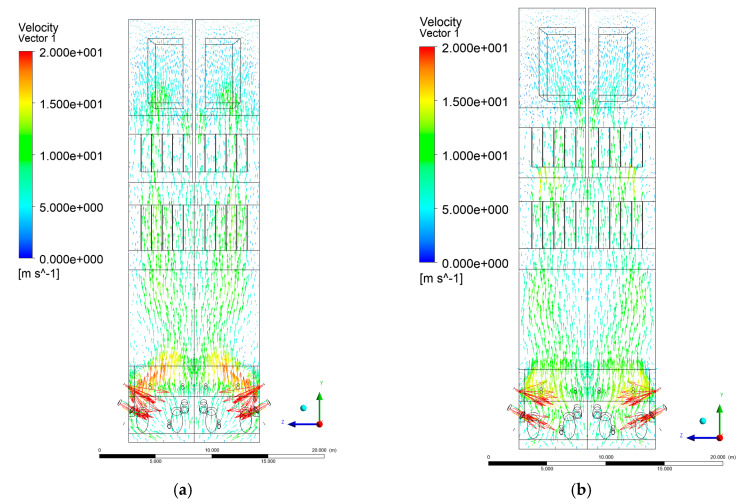
Gas velocity profiles, sectional view Y-Z-3, (**a**) Variant K2, (**b**) Variant K3.

Figure 6, Figure 7, Figure 8 and Figure 9 show the velocity distributions of the reactive gas mixture in the computational domain. In reference case K0 (Figure 6a), the velocity varied in the range 4 to 12 m/s, excluding the areas near individual air inlets, due to significant disturbances. The sectional views X-Y allow noticing of slight flow turbulence, which results mainly from the spatial configuration of air nozzles.

The velocity increase in each of the examined cases is observed in the platen superheater zones, corresponding to the reduced cross-sectional area. Such conditions favored the erosion process in this part of the furnace [21]. The flow characteristics were mainly the result of air and gas flows and negative pressure in the upper part of the combustion chamber. The gas supply affected, to some extent, the velocity distribution in the calculation domain due to the direct physicochemical relationship of the reactive mixture with its local composition and temperature. The supply of additional syngas to the combustion chamber in Variants K1, K2, and K3, caused the manifestation of additional gas sources in the combustion chamber. Moreover, the addition of dioxide-rich fuel gas led to an increase in temperature inside the furnace and CO_2_ concentration inside the lower part of the combustion chamber.

Figure 8 and Figure 9 show data on Y-Z cuts indicated upward flow direction, and moderate velocity in the central part of the furnace in each of the examined cases, which is consistent with the real behavior of the system, and allows us to state that the model is fully applicable for case studies of heat transfer by convection and radiation within said domain. The modification of boundary conditions (including the composition of the mixture at the domain inlets) allows obtaining clear and unambiguous information on the influence of such changes on the thermodynamic state of the system. Therefore, it is possible to exclude the influence of instability (caused by the change of boundary conditions) in solving the continuity and momentum conservation equations.

### 3.4. Combustion Chamber Temperature Distribution

In the reference case K0, a slight anisotropy of temperature distribution was visible in the X-Y and Y-Z sectional views. Conditions were much more homogeneous along the X-axis of the system, while local temperature changes resulted from the stationary model application. In the considered sectional views, temperature of a fluidized bed varied in the range of 750–850 °C. Due to the nature of the boundary conditions of the primary air inlet (homogeneous mass flux), a narrow area of local, relatively low temperatures, close to the air feed point, could be reported. Thus, the calculated temperature profile, in comparison to the experimental one, was shifted down towards the grid. However, the local temperatures, consistent with the experimental data equal to ~850 °C, were also registered.

The X-Y cuts indicated a shift of the area with the highest combustion intensity slightly towards the rear wall of the boiler, which was undoubtedly related to the location of the fuel feeders in this zone. An increase in temperature above the fuel feeding zone was observed. The central feeders supply fuel, and due to the impact of air inflow (Variant K0) on the front wall of the boiler, it slightly deviated from the original flight path towards the sidewalls, which was also visible in gas velocity profiles. This interaction was essential when considering the syngas supplied by this burner, as the spatial air concentration changed. The results obtained for cut Y-Z-3 indicated that in Variant K0, the air supplied by two start-up burners closest to the coal feeders (sidewall) had a positive effect on combustion efficiency, which was expressed in higher-temperature fields in the mixing zone of fuel and air. Therefore, these airstreams were not considered for replacement with syngas in further calculations.

A slight decrease in temperature in the wall regions was demonstrated in each sectional view as an effect of bed-to-wall heat transfer, mainly due to high suspension density on the membrane-walls [22]. However, the higher solids concentration on the walls (inert material, fuel, ash after fuel burnout) resulted in slight convergence problems in the CFD model [23,24]. Despite the high density of the grid, its cells were still large, which means that there was a relatively significant distance between the wall and the first node closest to the wall, where it was necessary to interpolate the temperature profile [25,26]. In some cases, it manifested a local increase in temperature next to the walls (visible also in the section of platen superheaters). For a smaller scale of the system, a solution might be the use of a higher-density grid in the wall layer, which, nevertheless, always results in a significant increase in the total number of control cells and drastically extends the computational time [27]. These problems do not exist in zones of greater distance from the walls, so the discussed effect did not influence the obtained results.

Due to the local character of changes resulting from the modification in air and fuel supply, the temperature distributions in Variants K1–K3 were best demonstrated by the sections containing the start-up burners or adjacent areas, especially Y-Z-2 and Y-Z-3, as well as X-Y-4 or X-Y-5 sectional views.

The results demonstrate a significant influence of the syngas supply by the start-up burners on local temperature increase in the domain (Figure 10, Figure 11, Figure 12, Figure 13, Figure 14, Figure 15, Figure 16, Figure 17, Figure 18, Figure 19, Figure 20, Figure 21, Figure 22, Figure 23, Figure 24, Figure 25, Figure 26, Figure 27, Figure 28, Figure 29 and Figure 30). The effect is observed as reaction zones of flammable gas components at short distances from the inlet to the combustion chamber (e.g., sectional views Y-Z: Figure 13, and X-Y: Figure 23, Figure 24, Figure 25, Figure 26, Figure 27, Figure 28, Figure 29 and Figure 30).

The unreacted or partially reacted (CH4 → CO) syngas moved to the lower parts of the combustion chamber, where it reacted with primary air. It caused a local temperature increase and shifted down the combustion zone of solid fuel due to its temperature rise. The effect was visible in the form of a local temperature rise reaching and even locally exceeding 1000 °C. Such a situation may lead to de-fluidization due to the melting or fusion of bed solids [16].

Moreover, a significant reduction in oxidant access to solid fuel was demonstrated, despite the reduction in coal and being supplanted by syngas. Differences in the combustion kinetics in the gaseous (homogeneous) phase, and coal combustion (heterogeneous phase) resulted in the favoring of combustible gas in the global chemistry of the processes taking place in the combustion zone. This may be explained by the fact that the combustion of char is a two-step process comprising of oxygen transportation to the carbon surface and the reaction of carbon with oxygen on said carbon surface [28,29,30,31]. This effect is visible even in the case of such a simple combustion mechanism as that used in the developed CFD model.

It is also worth emphasizing that due to the relatively low complexity of the model, the analysis did not cover many additional aspects related to the condition changes in the combustion chamber. The issues of NO_x_ emissions directly related to the temperature distribution in the combustion chamber was also essential in the considered cases [32,33,34,35,36].

Therefore, it should be concluded that the discussed idea is not recommended for use in the considered CFB unit. The obtained results confirm the statement underlined in [6,37], that the fuel range of an individual boiler depends on various factors, including fuel properties and furnace design parameters.

### 3.5. Combustion Chamber Carbon Dioxide Concentrations

**Figure 31:**
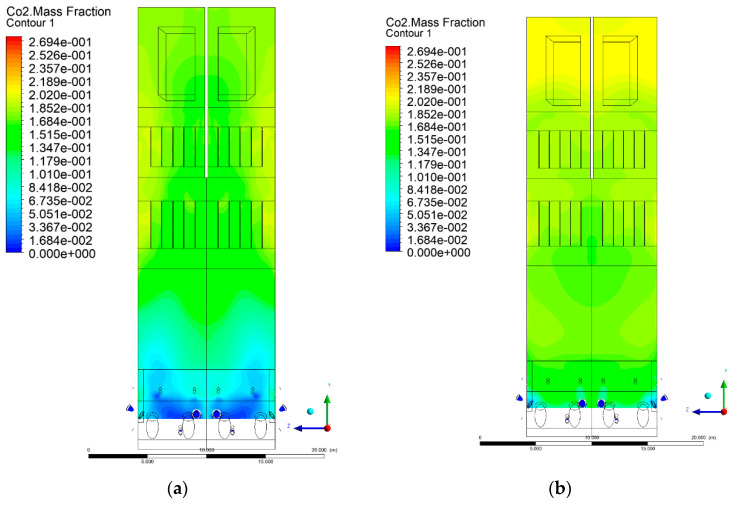
CO_2_ concentration in the chamber for sectional view Y-Z-1, (**a**) Variant K0, (**b**) Variant K1.

**Figure 32:**
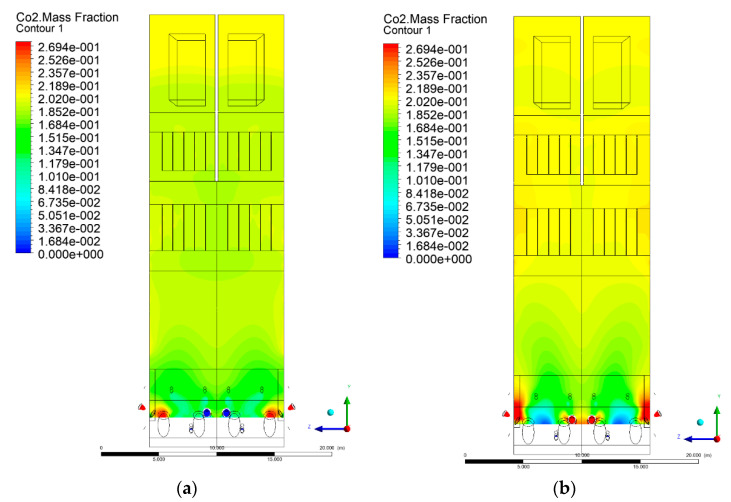
CO_2_ concentration in the chamber for sectional view Y-Z-1, (**a**) Variant K2, (**b**) Variant K3.

**Figure 33:**
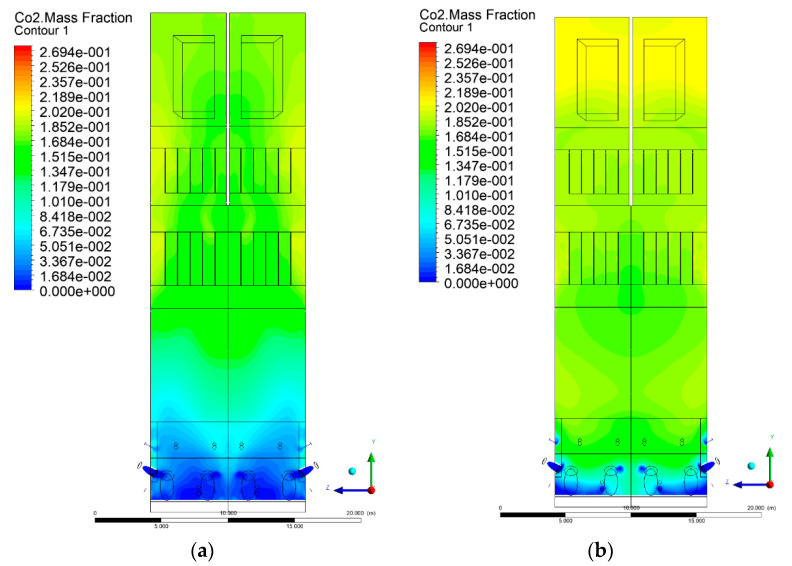
CO_2_ concentration in the chamber for sectional view Y-Z-2, (**a**) Variant K0, (**b**) Variant K1.

**Figure 34:**
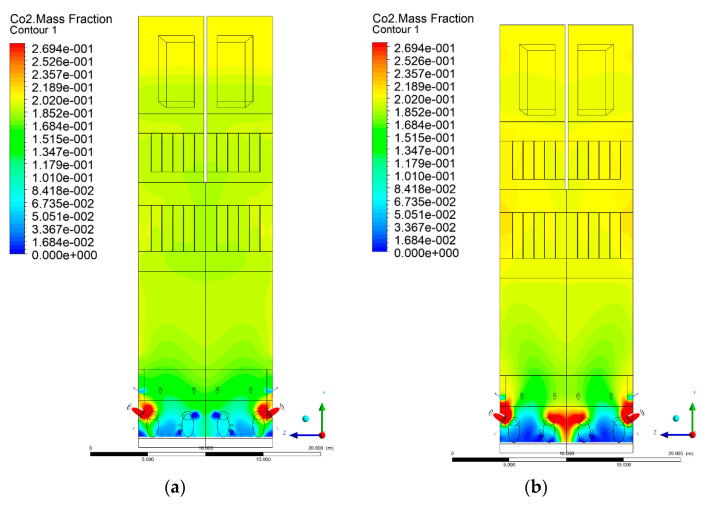
CO_2_ concentration in the chamber for sectional view Y-Z-2, (**a**) Variant K2, (**b**) Variant K3.

**Figure 35:**
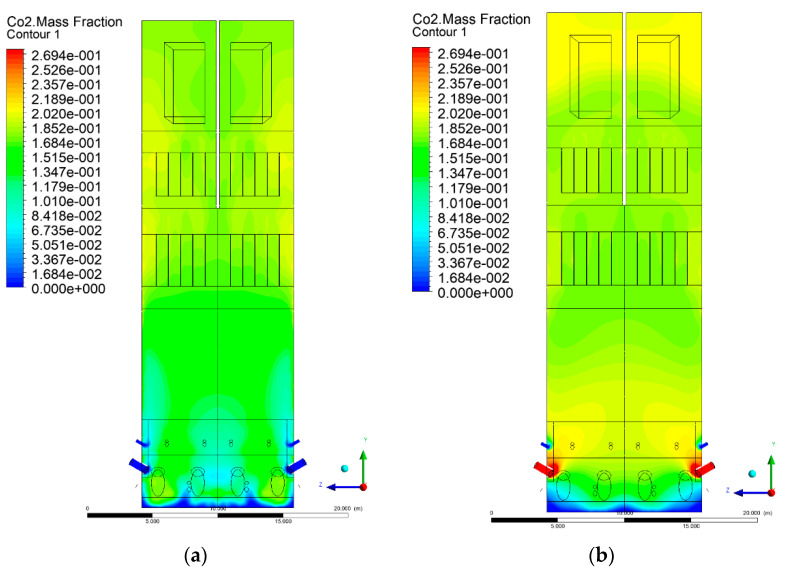
CO_2_ concentration in the chamber for sectional view Y-Z-3, (**a**) Variant K0, (**b**) Variant K1.

**Figure 36:**
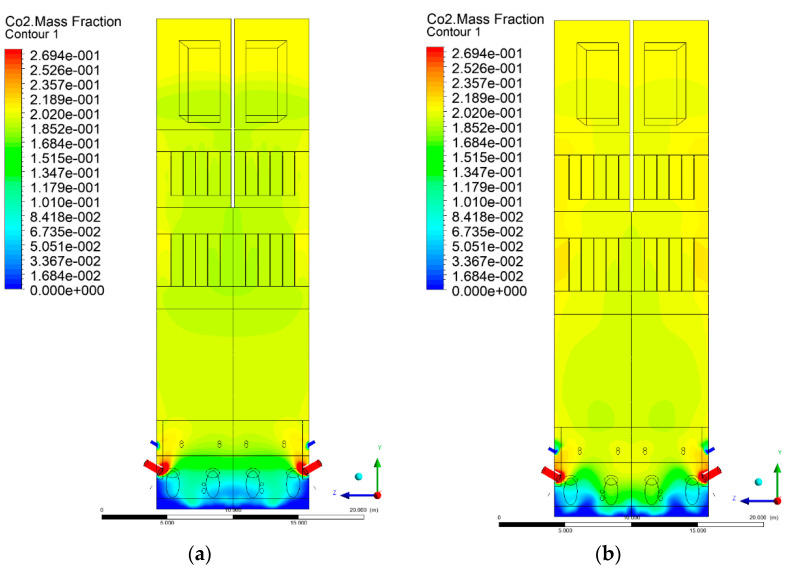
CO_2_ concentration in the chamber sectional view Y-Z-3, (**a**) Variant K2, (**b**) Variant K3.

**Figure 37:**
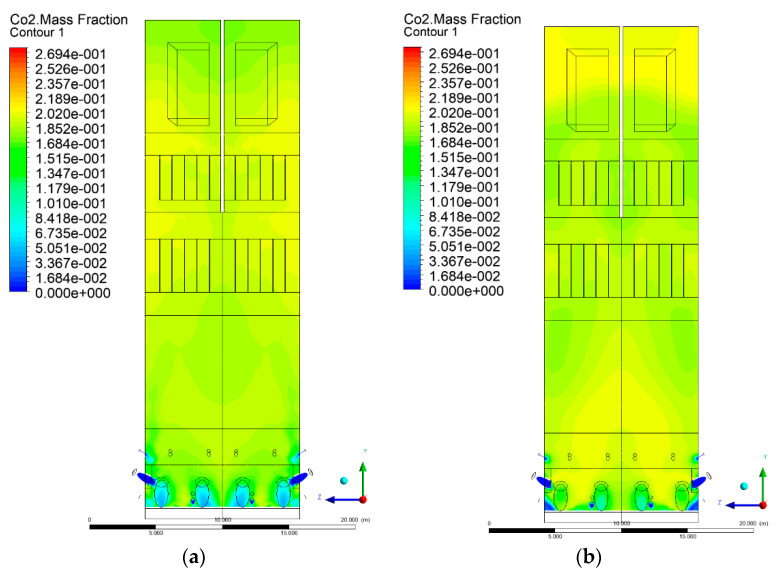
CO_2_ concentration in the chamber for sectional view Y-Z-4, (**a**) Variant K0, (**b**) Variant K1.

**Figure 38:**
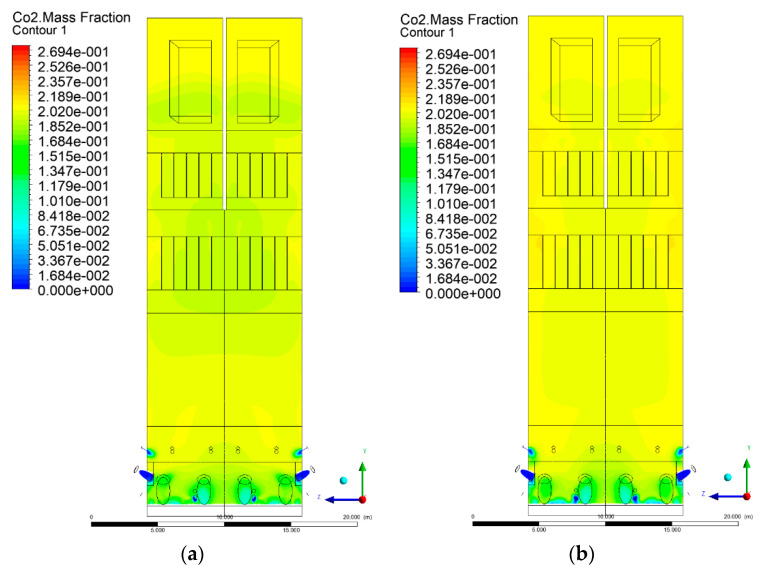
CO_2_ concentration in the chamber for sectional view Y-Z-4, (**a**) Variant K2, (**b**) Variant K3.

**Figure 39:**
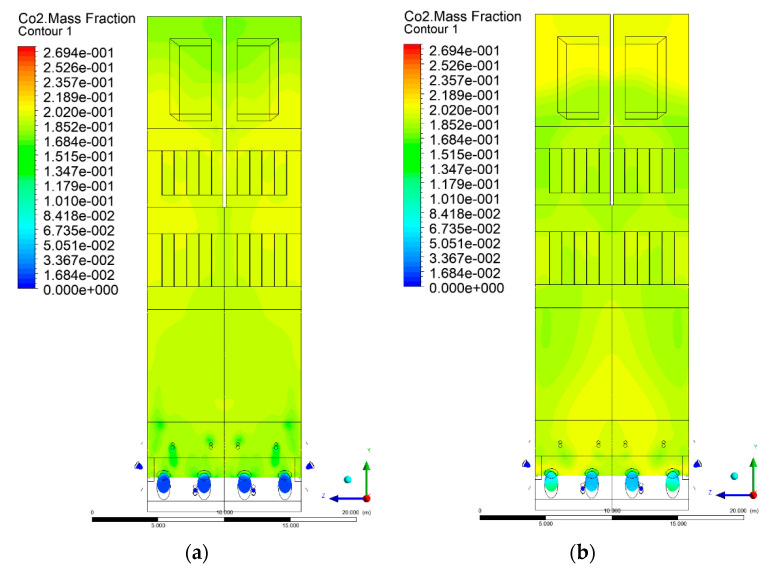
CO_2_ concentration in the chamber for sectional view Y-Z-5, (**a**) Variant K0, (**b**) Variant K1.

**Figure 40:**
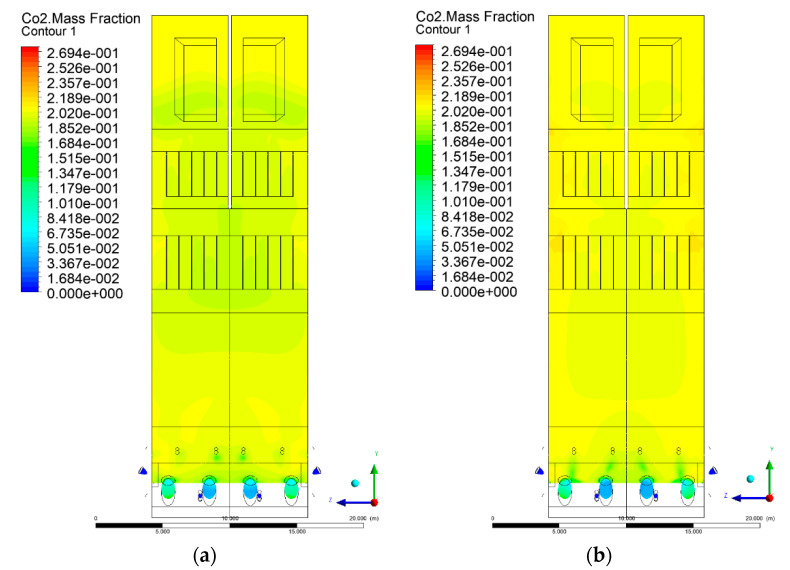
CO_2_ concentration in the chamber for sectional view Y-Z-5, (**a**) Variant K2, (**b**) Variant K3.

**Figure 41:**
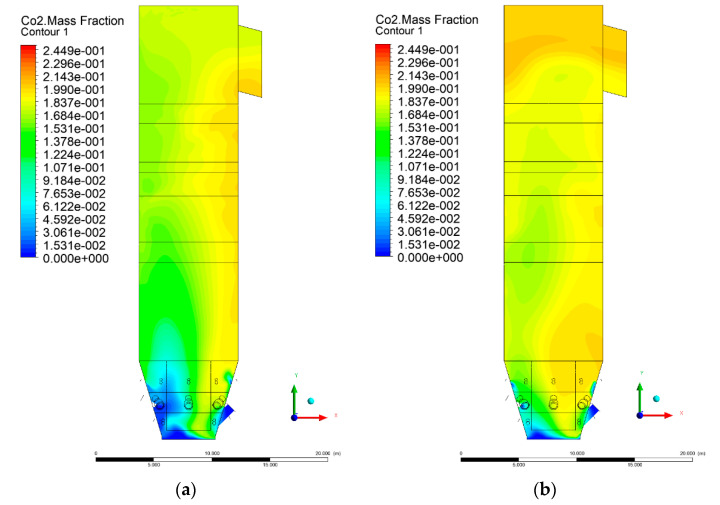
CO_2_ concentration in the chamber for sectional view X-Y-1, (**a**) Variant K0, (**b**) Variant K1.

**Figure 42:**
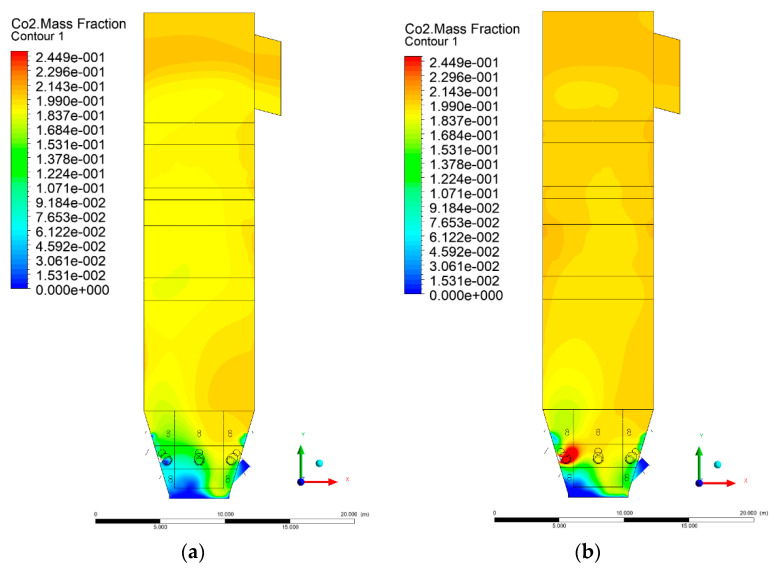
CO_2_ concentration in the chamber sectional view X-Y-1, (**a**) Variant K2, (b) Variant K3.

**Figure 43:**
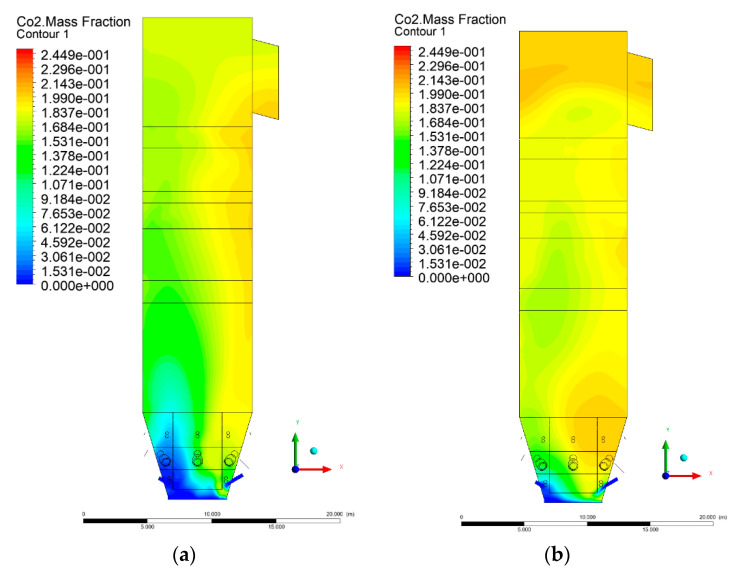
CO_2_ concentration in the chamber for sectional view X-Y-2, (**a**) Variant K0, (**b**) Variant K1.

**Figure 44:**
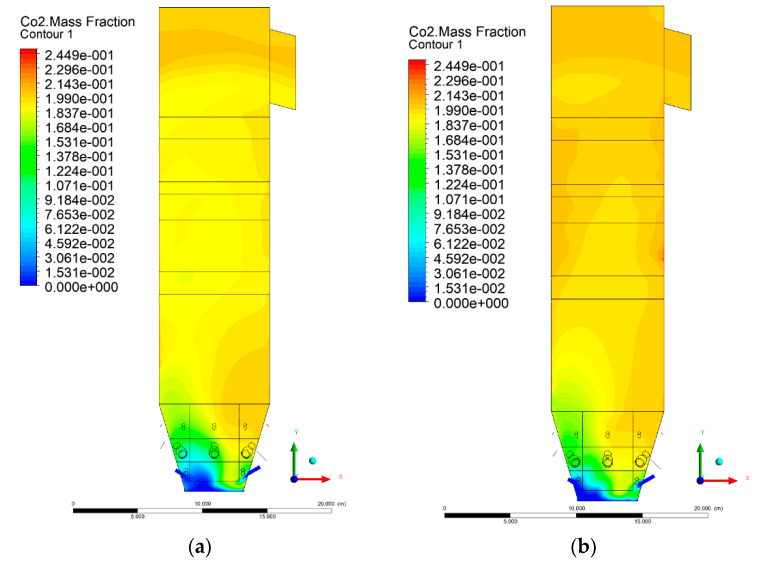
CO_2_ concentration in the chamber for sectional view X-Y-2, (**a**) Variant K2, (**b**) Variant K3.

**Figure 45:**
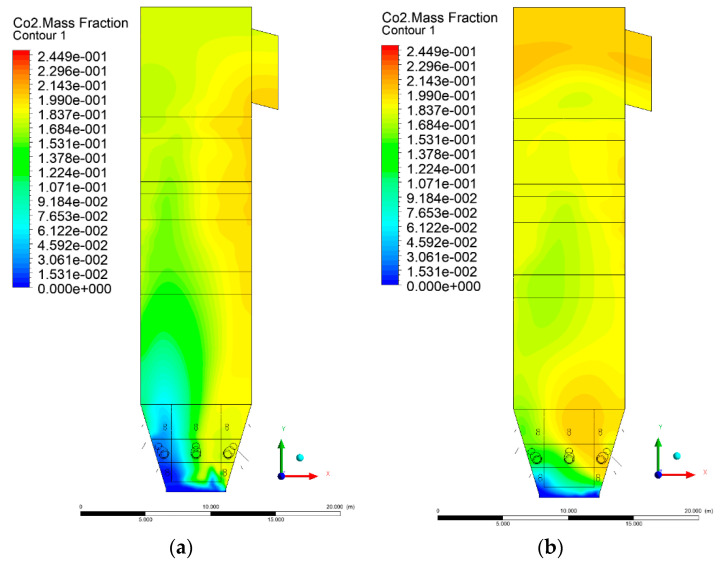
CO_2_ concentration in the chamber for sectional view X-Y-3, (**a**) Variant K0, (**b**) Variant K1.

**Figure 46:**
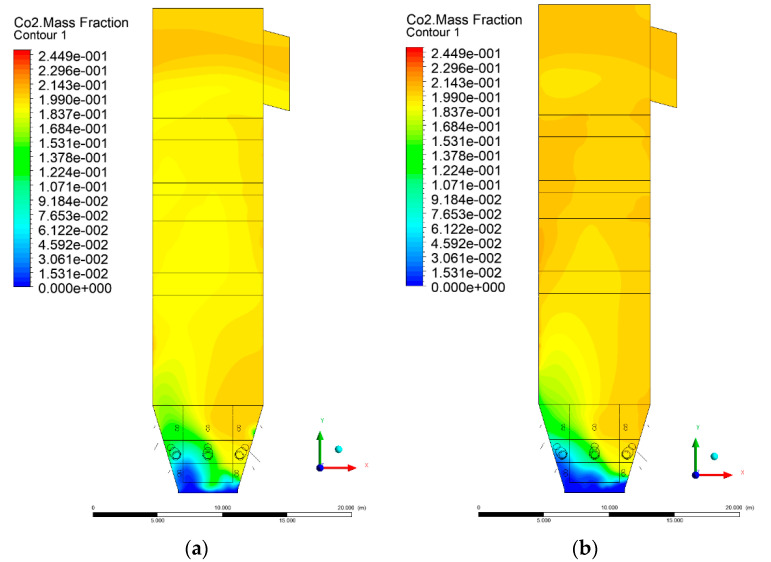
CO_2_ concentration in the chamber for sectional view X-Y-3, (**a**) Variant K2, (**b**) Variant K3.

**Figure 47:**
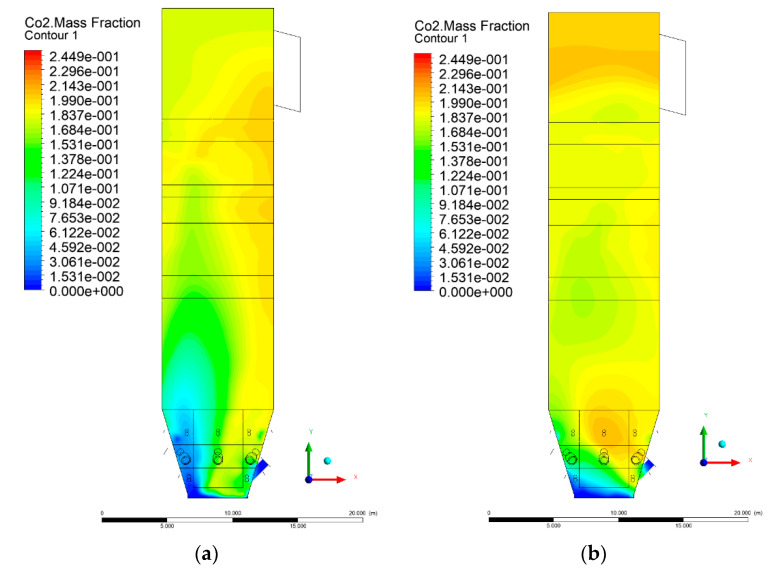
CO_2_ concentration in the chamber for sectional view X-Y-4, (**a**) Variant K0, (**b**) Variant K1.

**Figure 48:**
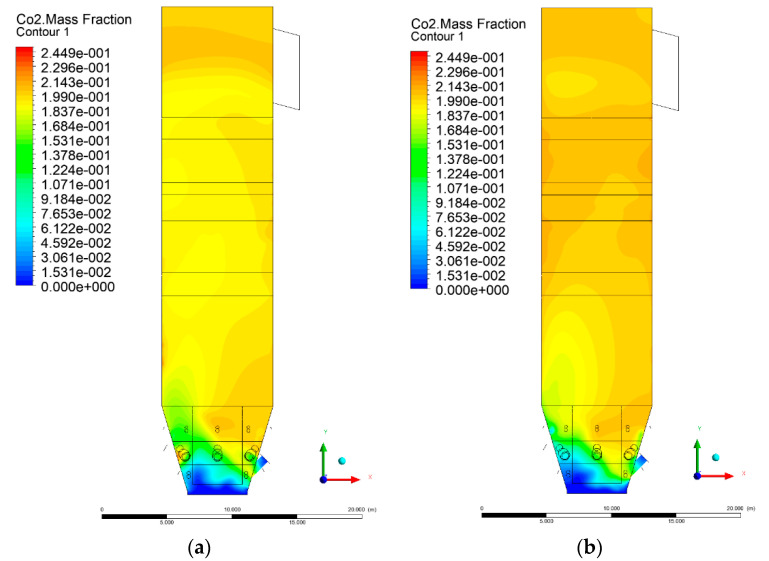
CO_2_ concentration in the chamber for sectional view X-Y-4, (**a**) Variant K2, (**b**) Variant K3.

**Figure 49:**
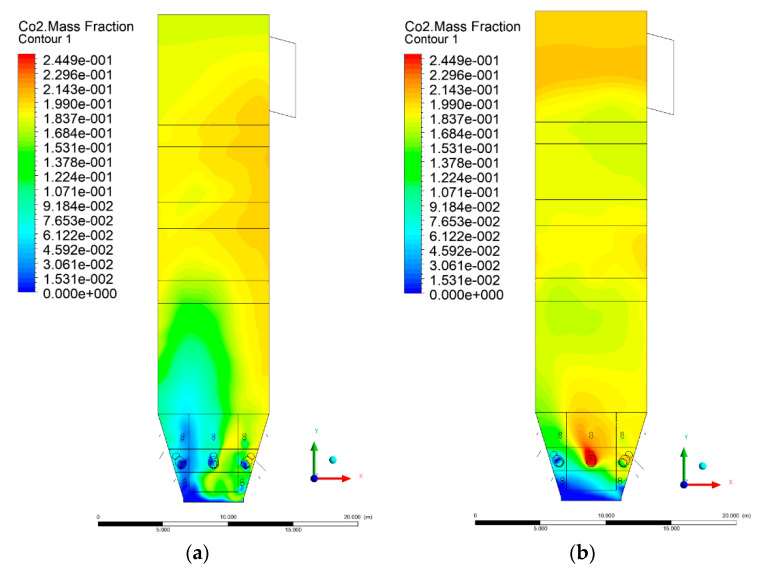
CO_2_ concentration in the chamber for sectional view X-Y-5, (**a**) Variant K0, (**b**) Variant K1.

**Figure 50:**
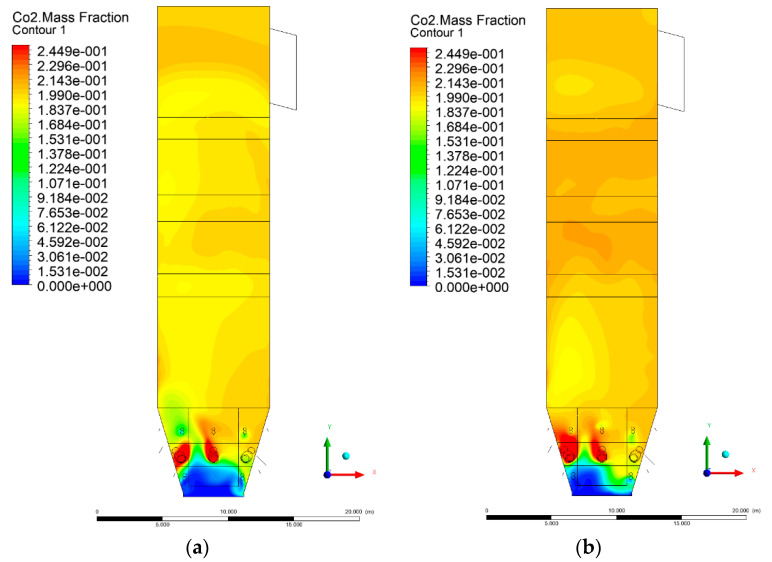
CO_2_ concentration in the chamber for sectional view X-Y-5, (**a**) Variant K2, (**b**) Variant K3.

Because the syngas supplied in the K1–K3 variants is rich in carbon dioxide, the concentration of this compound in the calculation domain increased in individual cases, not only due to the formation of additional combustion zones, but also through the distribution of CO_2_ from the gas flowing into the furnace. The effect was visible in the presented sectional views (Figure 31, Figure 32, Figure 33, Figure 34, Figure 35, Figure 36, Figure 37, Figure 38, Figure 39, Figure 40, Figure 41, Figure 42, Figure 43, Figure 44, Figure 45, Figure 46, Figure 47, Figure 48, Figure 49 and Figure 50). Due to different properties (specific heat, density, thermal conductivity) of the reactive mixture and the gas supplied, changes in local CO_2_ concentrations were observed, generating local zones rich in this compound.

The fields of CO_2_ concentrations in the furnace were closely related to the reactivity zones of the reactants in the calculation domain. Moreover, the fact of the participation of carbon dioxide molecules in the radiation heat transfer mechanism is also noteworthy. Being a compound absorbing radiation, CO_2_ influenced temperature distributions in the furnace [30,38,39].

## 4. Discussion and Concluding Remarks

Model research of a large-scale, OFZ-425, CFB boiler was carried out in this paper using a comprehensive 3D-CFD model. Models built using the CFD approach are the most comprehensive, numerically sophisticated, and advanced, based on detailed consideration of chemical kinetics and individual physical processes, gas, and solids continuity equations, momentum balances, and appropriate constitutive equations [2,40]. The purpose of the model was to identify the thermal-flow phenomena occurring in the fluidized bed under its standard operating configuration and modified conditions, i.e., with additional gaseous fuel supply. The influence of the introduced syngas on the distribution of pressures, temperatures, gas flow rate, and CO_2_ concentrations in the furnace of the CFB boiler was evaluated. This undertaking emerges from an awareness of the growing environmental pollution and climate change, and it constitutes another attempt to find a method to reduce emissions from fossil fuel combustion [41,42,43].

In the case of the mono-combustion case, the distribution of pressures inside the furnace was similar to the experimental data. All the discrepancies resulted from the simplifications in the defined hydrodynamic and reactions model. In the reference case K0, the flow velocity in the bed varied between 4 and 12 m/s. A significant velocity increase was observed in each of the examined cases in the zones of paten superheaters. The supply of additional syngas to the combustion chamber in Variants K1, K2, and K3, did not cause any changes in the pressure distribution and velocities compared to the reference case K0.

The syngas supply affected CO_2_ concentration profiles in the furnace. In all considered cases (K1, K2, K3), the CO_2_ concentrations in the combustion chamber increased, not only due to the formation of additional combustion areas, but also via the distribution of CO_2_ directly from the carbon dioxide-rich gas flowing into the furnace.

For each case (K1, K2, K3), the syngas supply led to an increase in temperature inside the furnace to over 1000 °C, mainly in the lower parts of the combustion chamber. Such a significant increase in temperature may lead to forming agglomerates, which can cause de-fluidization [16].

It is also worth emphasizing that due to the relatively low complexity of the model, the analysis did not cover many additional aspects related to the condition changes in the combustion chamber. The issues of NO_x_ emissions directly related to the temperature distribution in the combustion chamber was also essential in the considered cases.

The evaluation carried out in the paper clearly showed that the syngas supply into the furnace chamber of the OFz-425, CFB boiler is not advisable and may lead to frequent emergency stops of the boiler.

## Figures and Tables

**Figure 10:**
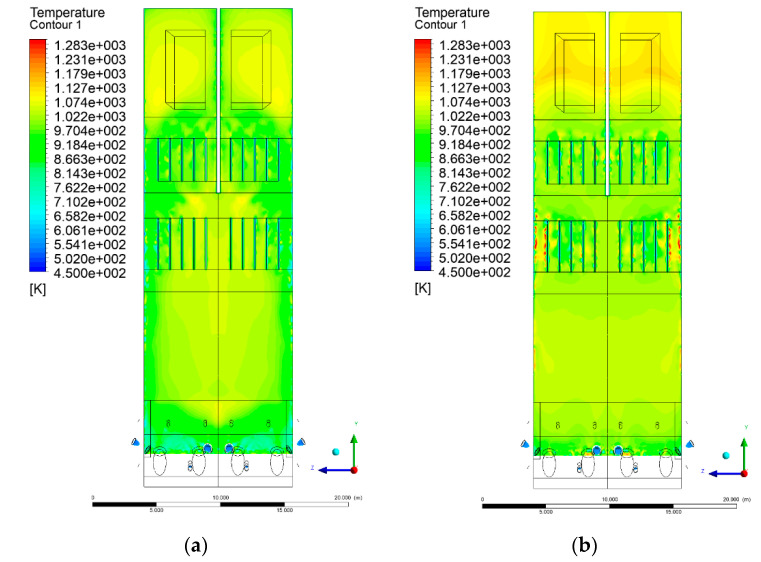
Temperature distribution, sectional view Y-Z-1, (**a**) Variant K0, (**b**) Variant K1.

**Figure 11:**
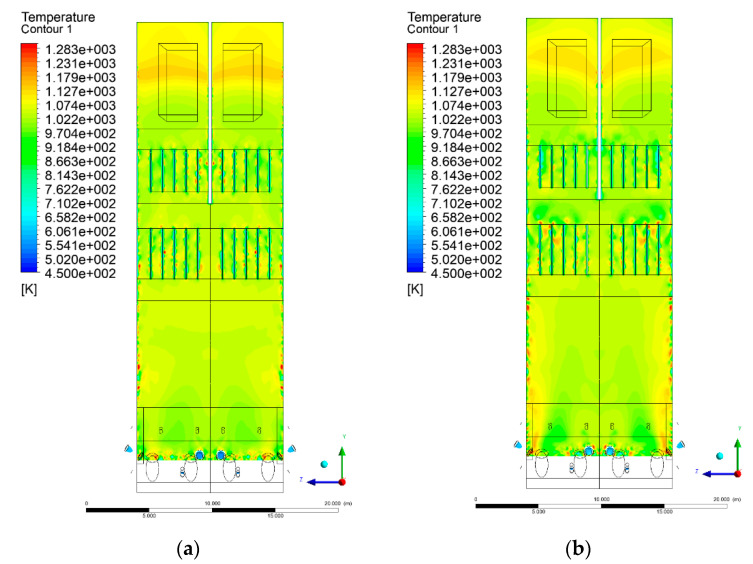
Temperature distribution, sectional view Y-Z-1, (**a**) Variant K2, (**b**) Variant K3.

**Figure 12:**
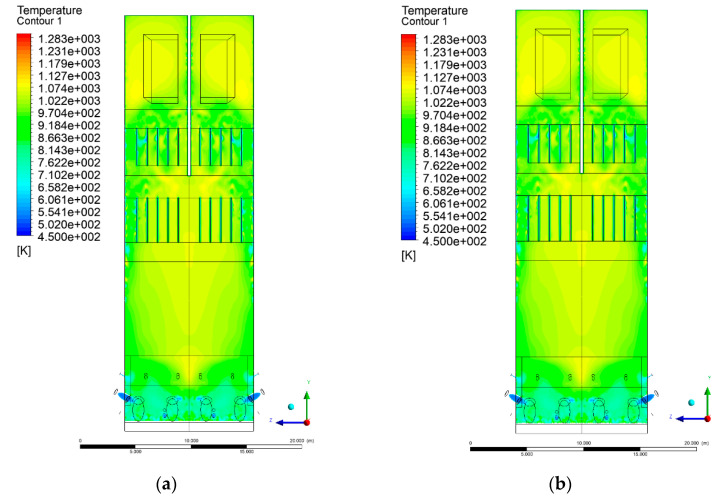
Temperature distribution, sectional view Y-Z-2, (**a**) Variant K0, (**b**) Variant K1.

**Figure 13:**
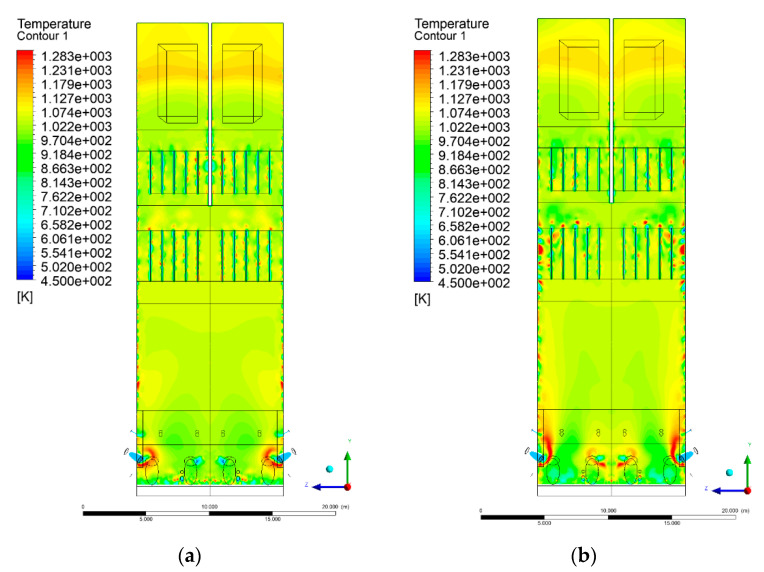
Temperature distribution, sectional view Y-Z-2, (**a**) Variant K2, (**b**) Variant K3.

**Figure 14:**
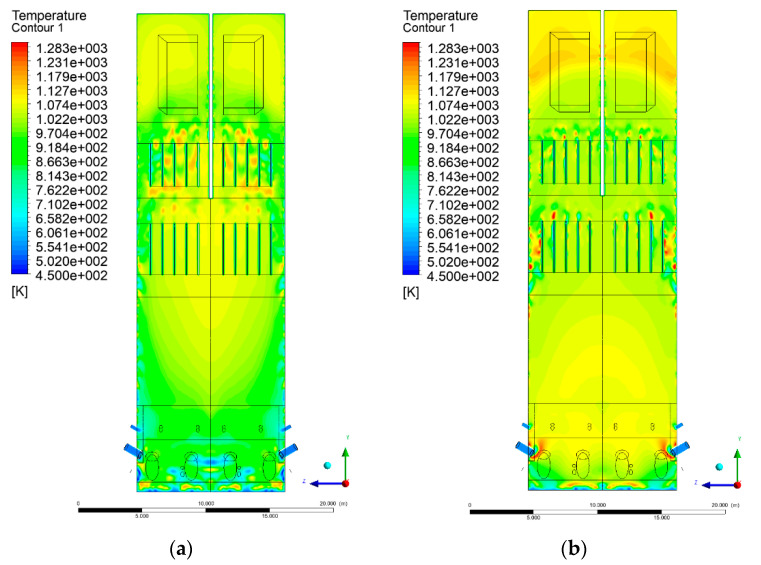
Temperature distribution, sectional view Y-Z-3, (**a**) Variant K0, (**b**) Variant K1.

**Figure 15:**
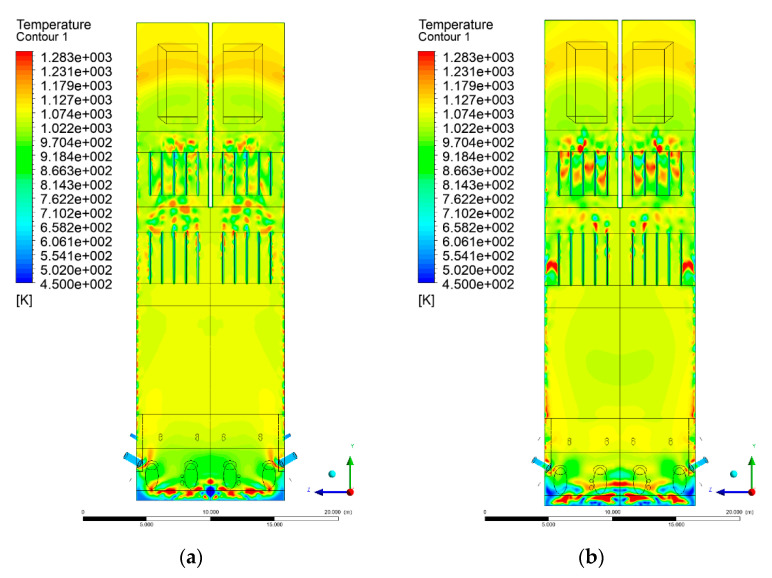
Temperature distribution, sectional view Y-Z-3, (**a**) Variant K2, (**b**) Variant K3.

**Figure 16:**
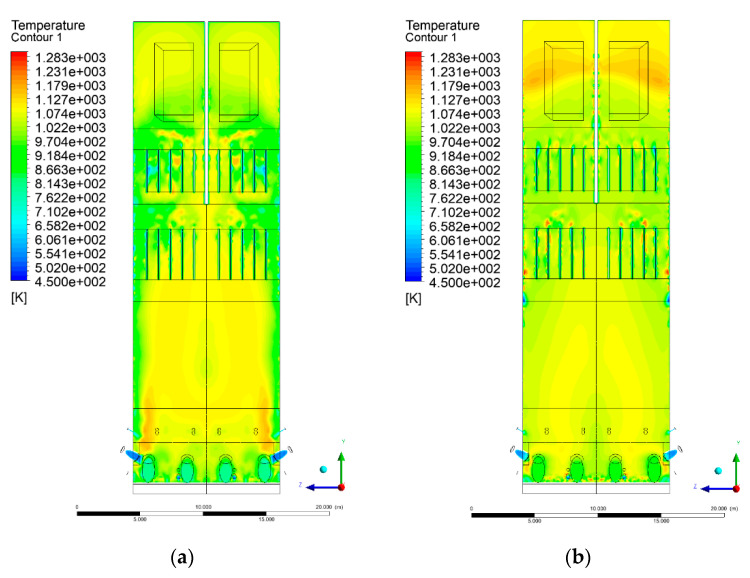
Temperature distribution, sectional view Y-Z-4, (**a**) Variant K0, (**b**) Variant K1.

**Figure 17:**
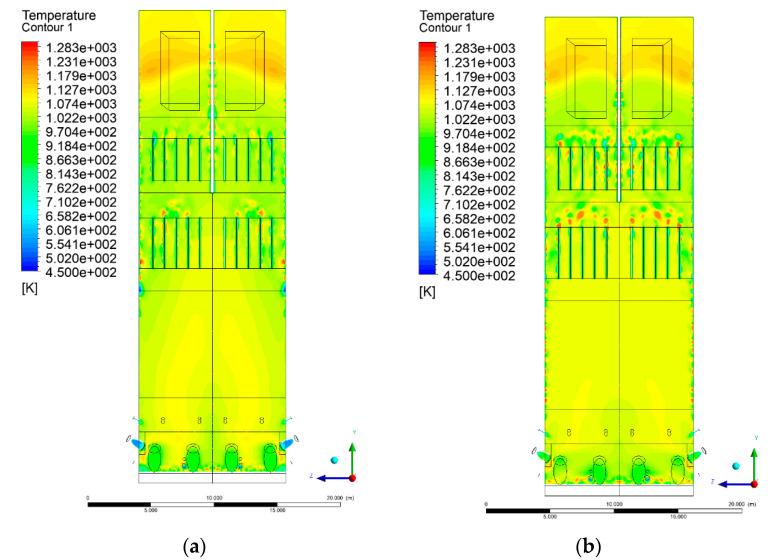
Temperature distribution, sectional view Y-Z-4, (**a**) Variant K1, (**b**) Variant K2.

**Figure 18:**
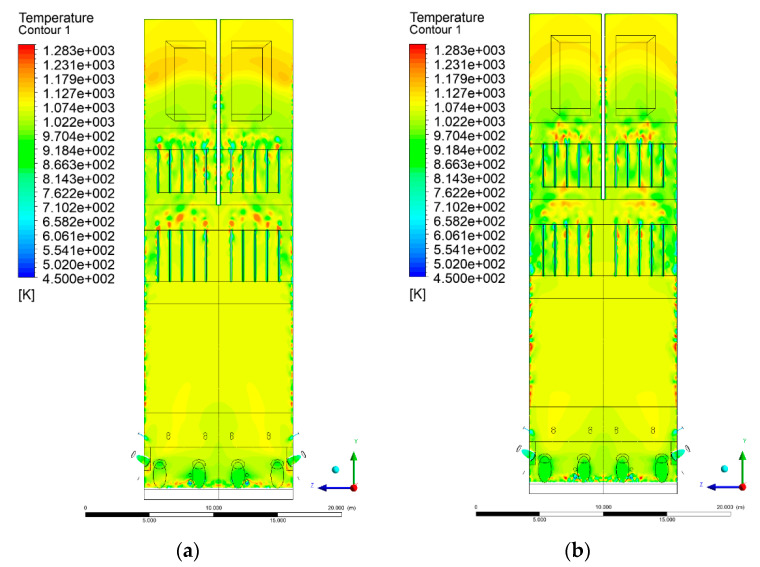
Temperature distribution, sectional view Y-Z-4, (**a**) Variant K2, (**b**) Variant K3.

**Figure 19:**
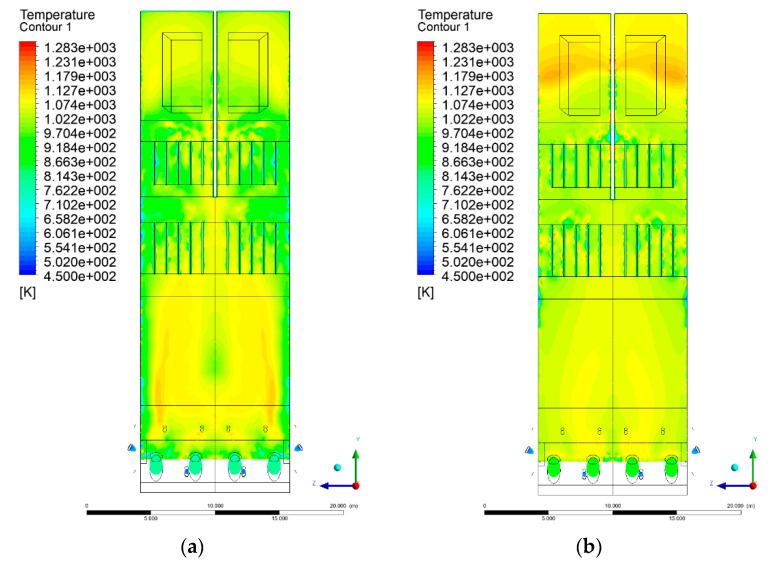
Temperature distribution, sectional view Y-Z-5, (**a**) Variant K0, (**b**) Variant K1.

**Figure 20:**
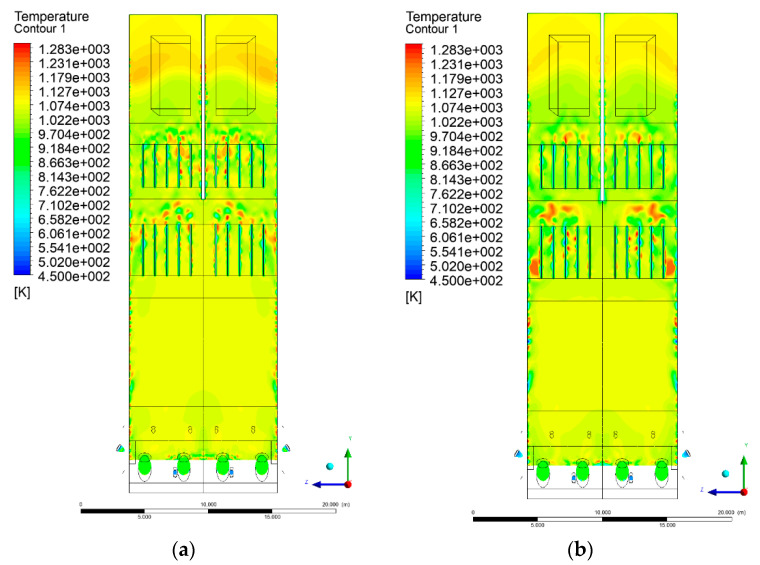
Temperature distribution, sectional view Y-Z-5, (**a**) Variant K2, (**b**) Variant K3.

**Figure 21:**
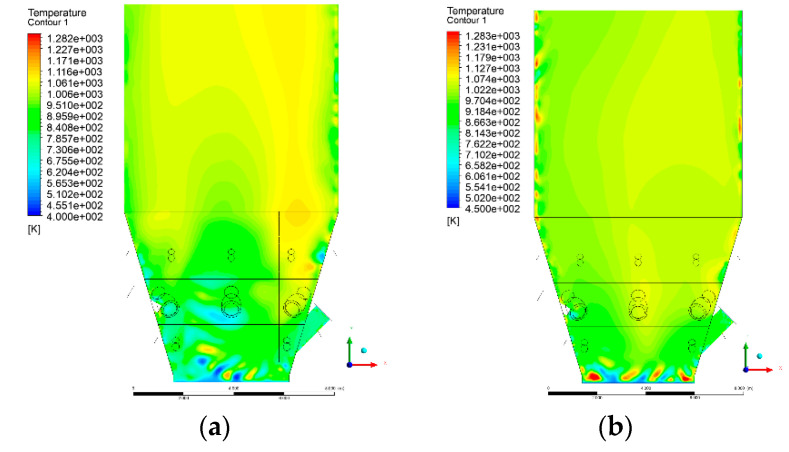
Temperature distribution, sectional view X-Y-1, Variants (**a**) K0 and (**b**) K1.

**Figure 22:**
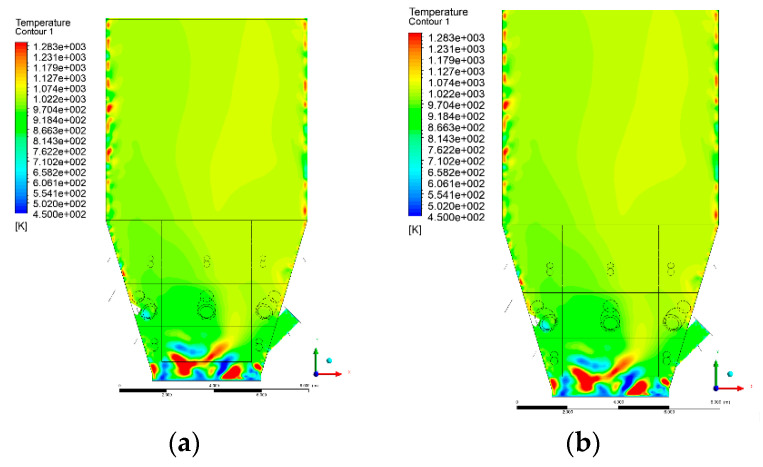
Temperature distribution, sectional view X-Y-1, Variants (**a**) K2 and (**b**) K3.

**Figure 23:**
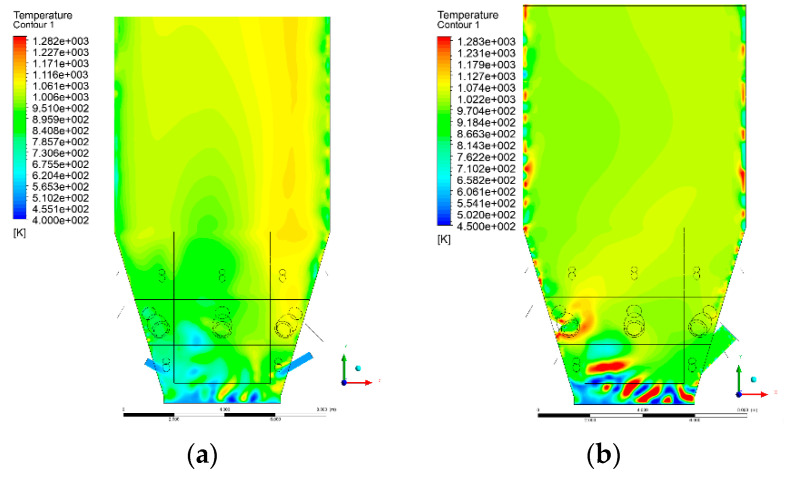
Temperature distribution, sectional view X-Y-2, Variants (**a**) K0 and (**b**) K1.

**Figure 24:**
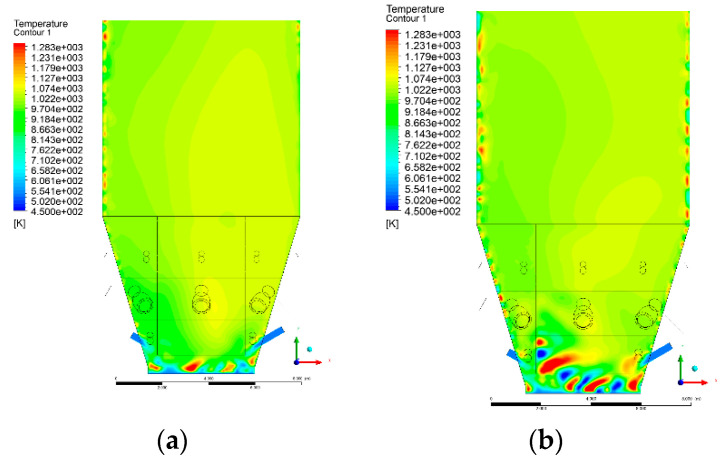
Temperature distribution, sectional view X-Y-2, Variants (**a**) K2 and (**b**) K3.

**Figure 25:**
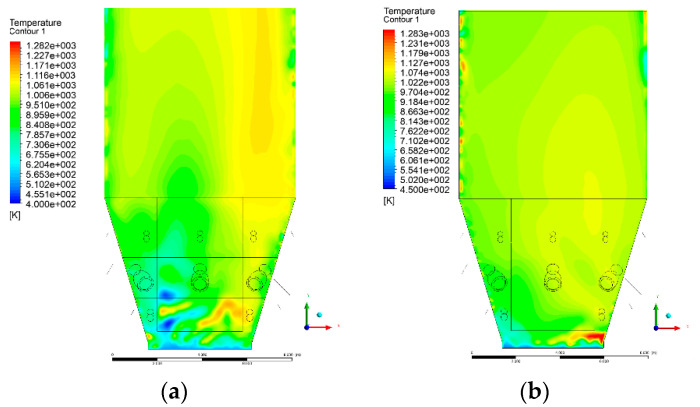
Temperature distribution, sectional view X-Y-3, Variants (**a**) K0 and (**b**) K1.

**Figure 26:**
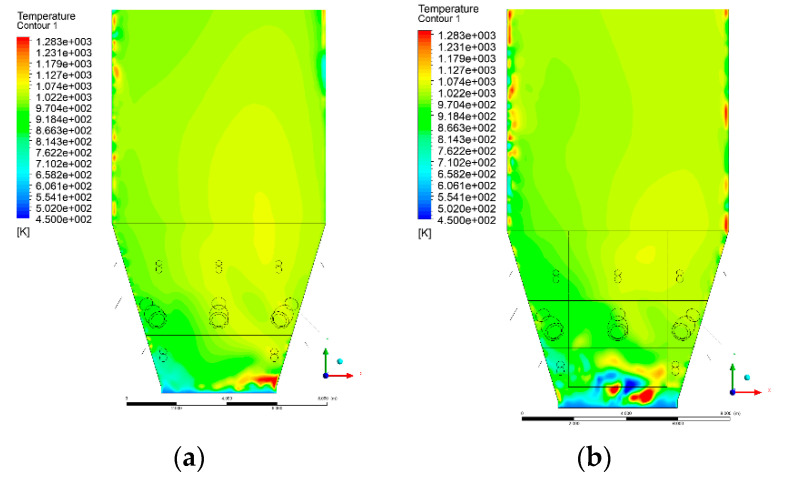
Temperature distribution, sectional view X-Y-3, Variants (**a**) K2 and (**b**) K3.

**Figure 27:**
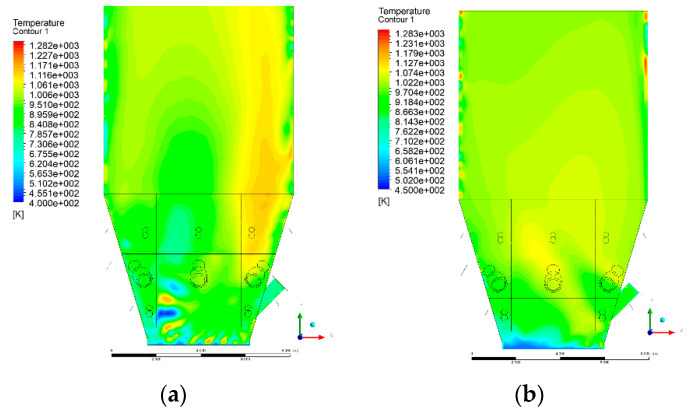
Temperature distribution, sectional view X-Y-4, Variants (**a**) K0 and (**b**) K1.

**Figure 28:**
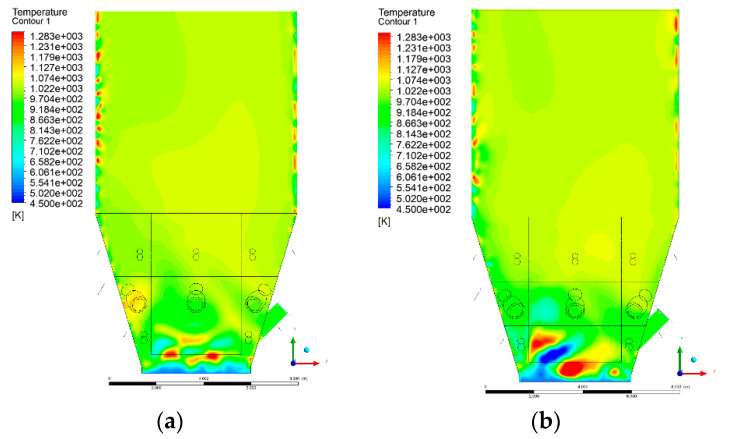
Temperature distribution, sectional view X-Y-4, Variants (**a**) K2 and (**b**) K3.

**Figure 29:**
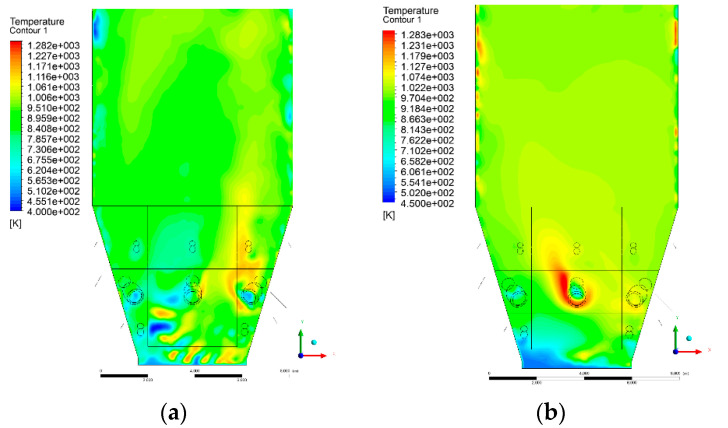
Temperature distribution, sectional view X-Y-5, Variants (**a**) K0 and (**b**) K1.

**Figure 30:**
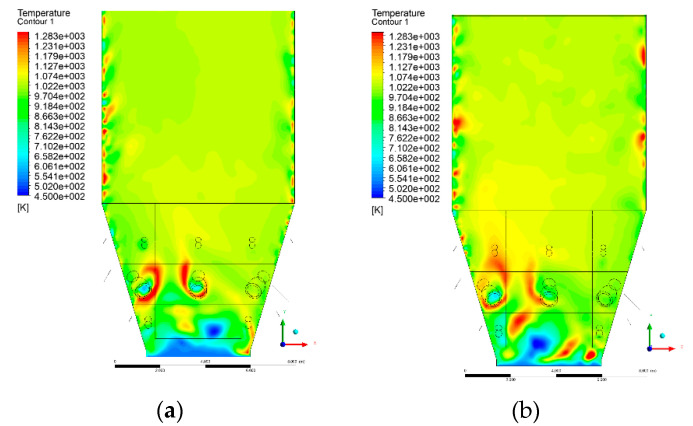
Temperature distribution, sectional view X-Y-5, Variants (**a**) K2 and (**b**) K3.

**Table 1:** Comparison of measured and calculated temperatures.

Height above the Grid m	Measured Temperature °C	Calculated Temperature °C	Relative Error %
0.2	834.1	859.1	−3.0
1.2	817.3	830.2	−1.6
6.0	779.2	795.9	−2.1

**Table 2:** Comparison of measured and calculated pressures.

Height above the Grid m	Measured Pressure kPa	Calculated Pressure kPa	Relative Error %
0.2	7.03	7.38	−4.99
1.2	2.57	2.70	−5.06
6.0	0.32	0.34	−6.25
